# The Complexity of Multiple Contraceptive Method Use and the Anxiety That Informs It: Implications for Theory and Practice

**DOI:** 10.1007/s10508-016-0706-6

**Published:** 2016-03-03

**Authors:** Lori Frohwirth, Nakeisha Blades, Ann M. Moore, Heather Wurtz

**Affiliations:** 1 Research Division, Guttmacher Institute, 125 Maiden Lane, 7th Floor, New York, NY 10038 USA; 2 Department of Sociomedical Sciences/Anthropology, Mailman School of Public Health, Columbia University, New York, NY USA

**Keywords:** Multiple method use, Contraception, United States, Qualitative

## Abstract

Despite clinical guidelines and national data describing the use of one contraceptive method as the best and most common way to prevent unintended pregnancy, limited evidence indicates a more complex picture of actual contraceptive practice. Face-to-face in-depth interviews were conducted in November of 2013 with a sample of women from two cities in the United States (*n* = 52). The interviews explored the ways participants used contraception to protect themselves from unintended pregnancy over the past 12 months. Most respondents reported using multiple methods, many of which are considered to be less-effective, within this timeframe. The practice of combining methods in order to increase one’s level of protection from pregnancy was prevalent, and was mainly enacted in two ways: by backing up inconsistent method use with other methods and by “buttressing” methods. These practices were found to be more common, and more complex, than previously described in the literature. These behaviors were mainly informed by a deep anxiety about both the efficacy of contraceptive methods, and about respondents’ own perceived ability to prevent pregnancy. These findings challenge prevailing assumptions about women’s contraceptive method use and have implications for clinical contraceptive counseling practice.

## Introduction

### Prevalence and Content of Multiple Method Use

Clinical practice recommendations in the U.S. state that women can best protect themselves from unintended pregnancy by using one effective method consistently and correctly (Centers for Disease Control and Prevention, 2013). Evidence from nationally representative surveys shows that most American women adhere to this advice (Jones, Mosher, & Daniels, 2012); national surveys of women in many other developed countries have yielded similar findings (Sato & Iwasawa, 2006; Spinelli, Talamanca, Lauria, & European Study Group on Infertility and Subfecundity, 2000; Toulemon & Leridon, 1998). However, other evidence suggests that, for some women, actual contraceptive practices may be more complex than indicated by these analyses, in that they may include more use of concurrent methods and less-effective^1^ methods (Brown et al., 2011; Frost, Singh, & Finer, 2007b; Gray, Chowdhury, Caldwell, & Al-Sabir, 1999; Horner et al., 2009; Jones, Lindberg, & Higgins, 2014; Parr & Siedlecky, 2007; Spinelli et al., 2000; Whittaker, Merkh, Henry-Moss, & Hock-Long, 2010). Understanding women’s true contraceptive practices is crucial, both for research that aims to describe contraceptive use in a population, and for clinical practice designed to reduce individual women’s risk of exposure to unintended pregnancy. The present study expands this understanding by qualitatively exploring the contraceptive behaviors of women from demographic groups most at risk of having an unintended pregnancy. Our detailed examination of our respondents’ contraceptive practices over a one-year timeframe revealed much more frequent and complex use of multiple methods than indicated by any previous work.

The picture of U.S. women’s contraceptive use evoked by the country’s preeminent survey of fertility behaviors, the National Survey of Family Growth (NSFG), found just 9 % of currently contracepting women reported using two or more methods in a given month, and small proportions of contracepting women using less-effective methods such as condoms (14 %), withdrawal (6 %), and Fertility Awareness-based Methods (FABMs, 2 %) within their method mix (Jones et al., 2012). Analyses in France, Denmark, Germany, Spain, and Japan yield comparable results (Sato & Iwasawa, 2006; Spinelli et al., 2000; Toulemon & Leridon, 1998). However, other evidence suggests that a higher proportion of women use multiple methods in their efforts to avoid unintended pregnancy. A few surveys of developed countries (U.S., Australia, Italy, and Poland) have found rates of multiple method use ranging from 23 to 44 % (Frost et al., 2007b; Parr & Siedlecky, 2007; Spinelli et al., 2000). A recent, nationally representative US study, specifically designed to capture multiple method use, found about one-third of women reporting multiple method use within the last 30 days (Jones et al., 2014).

A possible explanation for the discrepancy in these reported rates of multiple method use may be the underreporting of the use of less-effective methods. Jones et al. (2014) found approximately one-third of women reporting any withdrawal use and any condom use within the last month, findings they attributed to a questionnaire design that placed withdrawal at the top of the list of coital method choices. It is also possible that the use of FABMs may be underrepresented, due to them being used in tandem with other methods (Jaccard, 2009). In fact, closer examination of the women using multiple methods in some national surveys reveals that women who use less-effective methods are likely to be using them in combination with another method (Jones et al., 2014; Parr & Siedlecky, 2007; Sato & Iwasawa, 2006; Toulemon & Leridon, 1998).

### Mechanics and Motivations of Multiple Method Use

The literature above reported the existence of combination method use without shedding much light on exactly how or why women engage in this behavior. There are several documented ways, and reasons why, women may combine contraceptive methods or use more than one contraceptive method within the short timeframe typically examined (usually the past 30 days). One of the most commonly discussed forms is *dual use,* defined as concurrent use of a hormonal method to prevent pregnancy and condoms to prevent sexually transmitted infections (STIs) (Berer, 2006; Eisenberg, Allsworth, Zhao, & Peipert, 2012; Higgins & Cooper, 2012; Tyler et al., 2014; Woodsong & Koo, 1999). Method switching, i.e., the sequential use of multiple methods, for reasons such as experiencing side effects, changes in sexual frequency, ambivalence about preventing pregnancy, changing relationship dynamics, or a desire for a higher level of protection has also been extensively examined (Frost et al., 2007b; Grady, Billy, & Klepinger, 2002; Rosenberg & Waugh, 1998; Vaughan, Trussell, Kost, Singh, & Jones, 2008; Wellings et al., 2015).

A small number of studies have also documented the practice of substituting one method for another. In the Jones et al. (2014) study, of women who reported using either condoms or withdrawal during the month, 62 % reported switching between the two methods. Although that study did not explore the reasons why women substituted one method for another, other work has found that relationship characteristics have an effect on method choice; women with multiple partners may use condoms with certain partners and not others because of differing perceptions of the risk of STIs (Lansky, Thomas, & Earp, 1998; Lescano et al., 2006; Macaluso, Demand, Artz, & Hook, 2000).

Women’s and couples’ motivation to maintain some level of protection from pregnancy even when not using their primary^2^ method has also been shown to motivate method substitution. Whittaker et al.’s (2010) qualitative study of withdrawal use among young adult family planning clients revealed that respondents commonly substituted withdrawal for condoms when they did not have condoms on hand, when they were intoxicated, because of partner objections or when men desired more sexual pleasure. These findings are also present in other qualitative work examining the use of less-effective methods among specific populations or demographic subgroups (Gray et al., 1999; Horner et al., 2009). Method substitution in the form of “backing up” inconsistent pill use with other methods (Gold, 1999; Oakley, Potter, de Leon-Wong, & Visness, 1997; Weisman, Plictha, Nathanson, Ensminger, & Robinson, 1991; Williams-Deane & Potter, 1992) or using Emergency Contraception (EC) when a coital method was not used, was misused or failed has also been described (Abuabara et al., 2004; Keogh, 2005; Sanfilippo & Downing, 2008; Trussell, Raymond, & Cleland, 2014).

Another documented form of combination multiple method use, using methods concurrently at the same act of sex, also appears to be motivated in part by a desire to increase one’s level of protection from pregnancy. The Jones et al. (2014) study found that among respondents who reported using multiple methods within the same month, significant proportions reported using those methods during the same act of intercourse. Again, qualitative work on this topic provides some insight about motivations; a study in rural Bangladesh found that concurrent use of methods was driven by distrust of the efficacy of methods, a desire to increase the effectiveness of coital methods by combining them with use of “safe periods” or FABMs, a preference for ejaculation outside of the body and, among male respondents, uncertainty that their female partners were taking contraceptive pills properly (Gray et al., 1999). All of these reasons were echoed by Whittaker et al.’s (2010) respondents with respect to withdrawal. The combination use of withdrawal and/or condoms to boost the efficacy of FABMs during perceived fertile periods has been documented in other developing countries (Arevalo, Jennings, Nikula, & Sinai, 2004; Sinai & Jennings, 2014) and in at least one qualitative U.S. study of African American and Latina FABM users—even though official descriptions of FABMs only recommend periodic abstinence during the fertile days of the cycle (Jennings & Burke, 2011; Guzman, Caal, Peterson, Ramos, & Hickman, 2013).

### The Need for a Greater Understanding of Multiple Method Use

The existing research on the use of multiple contraceptive methods in combination is somewhat inconsistent, and work on this topic within the U.S. is limited. Varying rates of multiple method use have been found, with the bulk of the evidence showing that the practice is, at most, occurring among about a third of all women using contraception (Frost et al., 2007b; Jones et al., 2014; Mosher & Jones, 2010; Parr & Siedlecky, 2007; Sato & Iwasawa, 2006; Spinelli et al., 2000; Toulemon & Leridon, 1998). Such use is generally found to be concentrated among users of less-effective methods, although lower levels of combining less- and highly effective methods were also identified in these studies (Frost et al., 2007b; Gray et al., 1999; Jones et al., 2014; Parr & Siedlecky, 2007; Sato & Iwasawa, 2006; Spinelli et al., 2000; Toulemon & Leridon, 1998; Whittaker et al., 2010). There is some evidence that, in the U.S., both multiple method use and use of less-effective methods are higher among members of disadvantaged subgroups: young people and racial and ethnic minorities (Brown et al., 2011; Frost & Darroch, 2008; Guzman et al., 2013; Mosher & Jones, 2010; Sznitman et al., 2009). Reflecting this, in-depth research on combination and less-effective method use has either has been carried out among specific subgroups (rather than examining all women), or has focused on use of specific methods, rather than looking at a woman’s full range of contraceptive methods (Arevalo et al., 2004; Brown et al., 2011; Gray et al., 1999; Guzman et al., 2013; Horner et al., 2009; Sinai & Jennings, 2014; Sznitman et al., 2009; Whittaker et al., 2010). These limitations make it difficult to identify the full scope of combination multiple method use, while the variety shown in the existing literature on the prevalence, content, mechanics, and motivations of such use indicate that it is a fruitful area for further inquiry. The current study was designed to thoroughly explore women’s efforts to protect themselves from pregnancy by qualitatively examining contraceptive use over 1 year.

## Method

### Participants

We conducted 52 semi-structured face-to-face in-depth interviews (IDIs) with unmarried, lower- and middle-3 income women between the ages of 18 and 30 who had been sexually active with a man within the past year. The sample was selected to reflect the demographic groups that have been shown to have difficulty using contraception consistently and correctly, and who experience the highest rates of unintended pregnancy and abortion in the U.S. (Finer & Zolna, 2014; Frost & Darroch, 2008; Frost, Singh, & Finer, 2007a; Jones, Finer, & Singh, 2010; Mosher & Jones, 2010). We selected approximately equal numbers of African American, Latina and white women, as their contraceptive use patterns differ (Frost et al., 2007a; Frost & Darroch, 2008; Mosher & Jones, 2010). Additionally, we included women whose self-identified primary language was Spanish, in order to have representation in the sample from the largest immigrant group in the U.S. (Migration Policy Institute, 2014).

We recruited women in a large Northeastern city and a smaller Midwestern city through a professional recruiting company and Craigslist (www.craigslist.org), a popular classified advertisement website. In both locations, the recruiting company identified potential respondents from their database who met the screening criteria. Respondents from Craigslist were screened for eligibility via phone and email. Interviews took place during November 2013 in private offices in the cities where respondents were recruited.

The interviewers (LF, NB, and HW) were trained in the informed consent process, the administration of the interview guide, and interviewing techniques. Verbal and written consent was provided by all participants. All participants received $100 cash as compensation. During the informed consent process, respondents were told that they could stop the interview at any time and could decline to answer any interview question, and that they would still receive full compensation if they chose to do either; no respondent ended the interview early or declined to answer an interview question. Interviews lasted between 45 and 120 min. At the conclusion of the interview, participants filled out a short questionnaire on their socio-demographic characteristics. Study protocols were approved by the Guttmacher Institute’s Institutional Review Board.

### Measure

The interview guide focused on women’s contraceptive use to prevent pregnancy over the last 12 months. This timeframe was chosen in order to explore a longer period than most quantitative analyses and therefore potentially capture method switching (Jones et al., 2014; Mosher & Jones, 2010; Sato & Iwasawa, 2006; Toulemon & Leridon, 1998), but also to limit the possible recall biases and inability to recount details that may appear in work that asks respondents to report on lifetime contraceptive use (Horner et al., 2009; Reed, England, Littlejohn, Conroy Bass, & Caudillo, 2014; Whittaker et al., 2010). The guide was pretested with eight respondents who met all of the eligibility criteria, and changes were subsequently made to improve question clarity. In order to help respondents think about all contraceptive options, they were shown cards with labeled pictures representing all known methods and behaviors that can be used to prevent pregnancy: sterilization, an IUD, an implant, an injection, oral contraceptive pills, a patch, the ring, EC, male and female condoms, a cervical cap, a diaphragm, a sponge, spermicides, withdrawal, calendar or FABMs, and abstinence (defined as the avoidance of penile–vaginal intercourse to avoid the risk of pregnancy and hereafter referred to as “temporary celibacy” to differentiate it from the periodic abstinence that may be a part of some women’s practices of FABMs). Participants were asked to separate out any methods they had ever used for any reason, and then to separate those used in the past 12 months. If women did not initially include withdrawal, FABMs, or EC, they were prompted about whether they had used these methods, using colloquial terms. We then asked a series of questions about how women used those methods in the previous year, which enabled us to assess consistency and correctness of use, reasons for use other than pregnancy prevention and respondents’ perceptions of method efficacy. Methods used with different partners and women’s perceptions of their partners’ attitudes toward contraception were also explored. When more than one method was used over the course of the year, we probed about if and how the methods were used in conjunction with each other. Instances as well as periods of nonuse were also scrutinized.

### Data Analysis

All of the IDIs were digitally recorded and transcribed verbatim. Identifying information was stripped during the cleaning phase. Interviews conducted in Spanish were professionally translated and the translations were checked for accuracy by the interviewer.

We followed Agar’s qualitative strategy of reading each woman’s interview in its entirety to understand each respondent’s comprehensive narrative (Agar, 1980). Respondents’ narratives of their full contraceptive practices unfolded across the entire interview; we summarized this information for each respondent in a standardized format for this analysis (Huberman & Miles, 1998). The use of quotes in this analysis is therefore spare relative to other qualitative work, and we rely more heavily on the summaries described above in order to illustrate the phenomena described in this paper. Respondents are identified using pseudonyms.

Cross-case analysis was conducted to identify themes and concepts, and to explore similarities and differences. We counted the number of contraceptive methods respondents had used and analyzed how they used these methods (Miles & Huberman, 1994). Additionally, we performed targeted coding on respondents’ method use and factors that informed their beliefs and decisions about that use (LeCompte & Schensul, 1999). Coding was performed by two members of the research team (LF and NB) after over 95 % inter-coder reliability had been established.

## Results

Respondents’ demographic characteristics are shown in Table 1. One-fourth were Latina (*n* = 21, 7 of whom were interviewed in Spanish), 18 were Black, and 13 were White. One-third (*n* = 15) had children. Most (*n* = 30) had never been married and were not currently cohabiting and most (*n* = 32) were low income. Only one woman in our sample explicitly wanted to be pregnant at any time during the past year. Two women in our sample reported becoming pregnant during the year. Neither desired to become pregnant when she did; they both attributed their pregnancies to sexual encounters during which withdrawal was supposed to be used, but their partners failed to withdraw.

**Table 1:** Characteristics of in-depth interview respondents

	Total sample (*n* = 52)	% of total
Age		
20–25	27	52
26–30	25	48
	52	100
Race^a^		
White, nonhispanic	13	25
Black, nonhispanic	18	35
Hispanic	21	40
	52	100
Marital status		
Never married	30	58
Cohabiting	20	38
Separated	1	2
Divorced	1	2
	52	100
Language of interview		
English	45	87
Spanish	7	13
	52	100
Education		
9–11th	2	4
High school/GED	7	13
Some college	26	50
College graduate	17	33
	52	100
Parity^b^		
0	31	67
1	6	13
2	5	11
3+	4	9
	46	100
Income		
Under 200 % FPL	32	62
Between 200 and 300 % FPL	20	38
	52	100
Number of methods used in the last 12 months		
0	1	2
1	3	6
2	15	29
3	16	31
4	11	21
5	3	6
6	3	6
	52	100

*FPL* federal poverty line

^a^Some Hispanic women indicated race and ethnicity (i.e., White and Hispanic) and some only indicated Hispanic. Therefore, women indicated as Hispanic in this tabulation may also have indicated any or no race

^b^Six women did not provide a response to this question

Nearly all hormonal methods were reported in our sample: oral contraceptive pills (*n* = 22), the injectable (*n* = 6), IUDs (*n* = 5), contraceptive rings (*n* = 3), and contraceptive patches (*n* = 1). Ten women also reported using EC. Condoms (*n* = 38) were the most popular barrier method, while three women used spermicides (gel or film) with or without condoms. No other barrier methods were used. Traditional, behavioral method use was reported by more than 8 in 10 of our respondents. Withdrawal was the most commonly used method in our sample (*n* = 42). There was also widespread reported use of FABMs (*n* = 18) and temporary celibacy (*n* = 10).

The majority of respondents (48 of 52) reported using more than one contraceptive method in the previous 12 months. Approximately one-third of women used two methods within the year (*n* = 15), one-third reported three methods (*n* = 16), and the remaining third reported four (*n* = 11) or five or six methods (*n* = 6). Only one woman reported using no methods to prevent pregnancy in last year. The narratives of the multi-method users reveal various motivations affecting their contraceptive use, including sexual pleasure, partner preferences, experiencing negative side effects, health issues, and difficulty accessing methods. Since the desire to increase their perceived level of protection from pregnancy motivated the multiple method use of nearly all (43 of 48) of the women in our analytic sample, this paper examines the modes of combination, or “mechanisms,” most closely associated with this motivation.

**Fig. 1:**
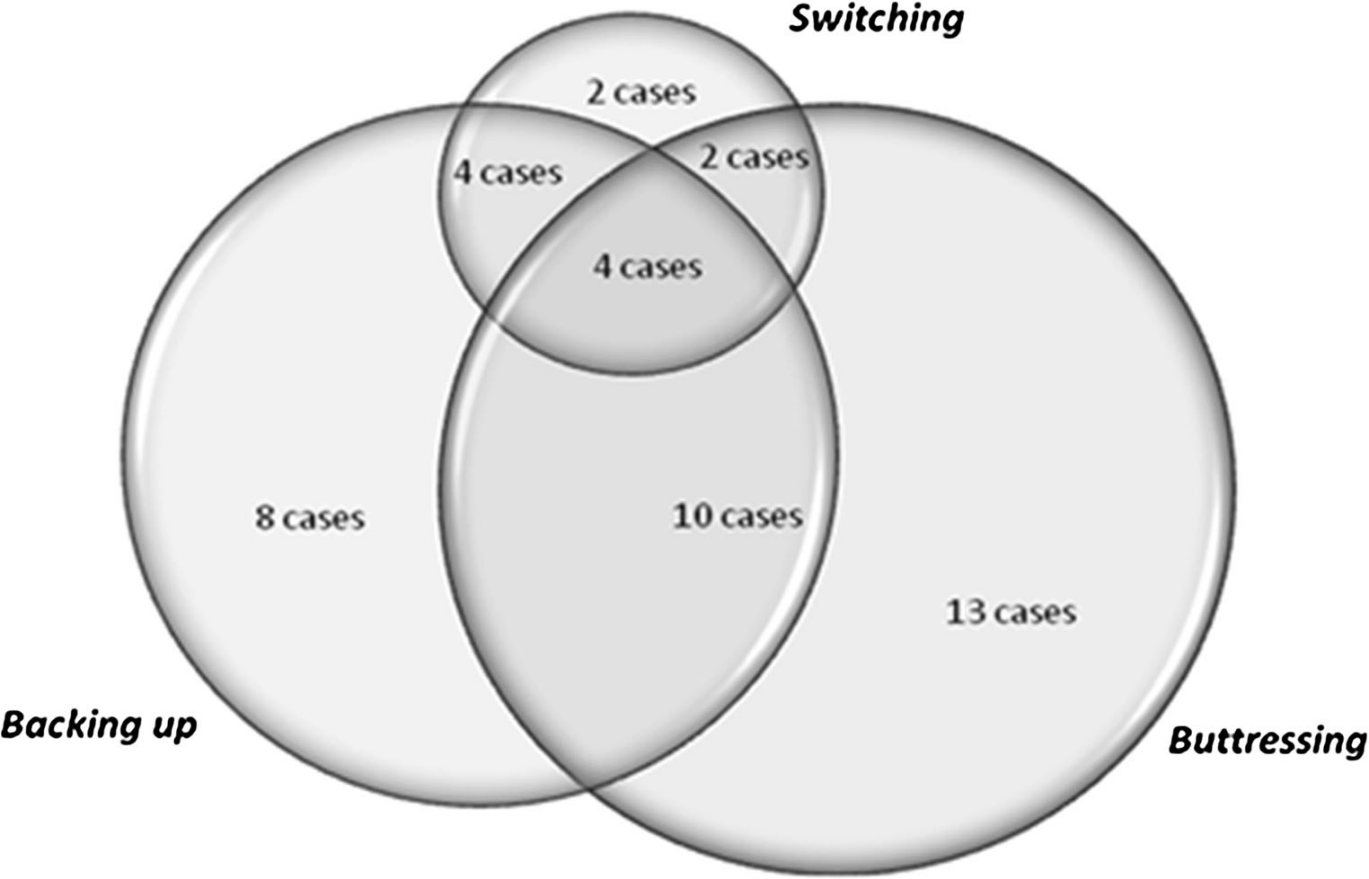
Forms of multiple method use among women seeking to increase protection from pregnancy, *n* = 43

Twelve women reported switching to a method that they perceived as more effective in order to increase their level of protection from pregnancy. As this type of multiple method use has been extensively documented, we will focus on two other mechanisms of combining methods motivated by a desire to be more protected from pregnancy that emerged as both more common and more complex than previously documented in the literature: backing up inconsistent method use and “buttressing” of methods (i.e., using two methods simultaneously to produce a perceived higher level of protection from pregnancy than one method used alone). Many (*n* = 20) of our respondents’ contraceptive strategies over the last 12 months encompassed more than one of these mechanisms (see Fig. 1).

### Backing Up Inconsistent Method Use

More than half of multiple method users in our sample (*n* = 26) described inconsistent use of a method backed up by the use of another method in order to maintain protection from pregnancy. Some of these respondents reported backing up their primary method, i.e., they occasionally substituted withdrawal for condoms, used another method to account for inconsistent pill use, and/or used EC when they had not used any other method at an act of intercourse.

However, our data reveal another level of backing up inconsistent method use: backing up a backup method with a tertiary method. Most often, the primary method was hormonal, the secondary method was condoms, and the tertiary was withdrawal. Sonya, a long-term pill user, reported that she often took her pills late or missed them up to 2 days in a row. As a result, she felt she needed to back up her pill use with another method, and preferred to use condoms for this purpose because she felt they would be most effective. However, Sonya’s condom use was inconsistent as well. Pressure from her partner, coupled with the fact that Sonya herself preferred withdrawal when she was intoxicated, led her to back up her condom use with withdrawal. Sonya reported that because her pill use was so erratic, she backed it up by using either condoms or withdrawal at most acts of sex over the past year, thereby implementing a three-layered system of substitution to achieve her desired level of protection from pregnancy.

EC was also named by many women as a tertiary backup when they were unable to enact their preferred substitution for their primary method, or when they when they were not confident about their secondary backup method. Audrey frequently either forgot to take her pills or took them late, and would use withdrawal to back up these missed pills. She did not try to use withdrawal at every act of intercourse though, and her partner was not always successful when they did intend to use it, so Audrey frequently took EC for additional protection. She reported taking EC six or seven times over the course of the year.

Women also mentioned experiencing gaps in prescription method use due to lost insurance, missed appointments, or other difficulties with access, and reported filling those gaps with a series of backup methods:

[R]espondent: […] When I went to get my refill [of pills], they told me that the insurance was canceled. […]

[I]nterviewer: So, [you said that] during the two weeks in which you weren’t taking [the pill], you used condoms and abstinence [temporary celibacy]. Why is that?

R: Because […] we are trying to do it [have sex] as little as possible, and we’re using this [condoms] when we do it. – Adrienne

Similarly, when Rosemary missed her appointment for her injectable and was waiting until she could get another one, she used temporary celibacy as a backup, and withdrawal as a tertiary backup when she had sex. In one instance during that period, her boyfriend did not withdraw on time. Frightened (but not impregnated) by this event, Rosemary switched to an IUD.

When explaining their reasons for inconsistent use that led them to back up their methods, our respondents reported lack of access to their primary method (e.g., running out of condoms or leaving home without their pills), intoxication, and pressure from partners. They also explained the way that their own sexual pleasure informed their practice of substituting withdrawal for condoms. They described using withdrawal in place of condoms when irritation from condoms interfered with their pleasure during sex, or when they wished to experience the pleasure they associated with sexual acts that included withdrawal. Faye reported alternating between condoms and withdrawal at most acts of sex, depending on “how passionate we’re feeling and how sensitive we’re feeling.” She clarified that withdrawal was more pleasurable for both of them, but she considered condoms to be more effective at preventing pregnancy, and so felt compelled to try to use them sometimes. Most women who alternated between condoms and withdrawal to maintain pregnancy protection while increasing sexual pleasure reported withdrawal as the more pleasurable option of the two methods, but this was not universal.

### Buttressing Method Use for More Perceived Protection From Pregnancy

Thirty women in our sample described a technique of using methods in combination, which we are naming “buttressing,” wherein one method is used with another in order to produce a perceived higher level of protection from pregnancy than from one method alone. As opposed to backing up, buttressing did not happen when women felt unprotected due to inconsistent use of a primary method. Rather, buttressing was the concurrent use of multiple contraceptive methods when women (or their partners) felt the need to layer on an additional method to bolster the effectiveness of a primary method.

Most instances of buttressing in our sample involved combinations of less-effective methods: condoms, withdrawal, and FABMs. Our data revealed the flexibility of this practice, in that the augmentation was not always based on a hierarchy from most-effective to least-effective, i.e., women buttressed less-effective methods with more-effective methods, but also buttressed more-effective methods with less-effective methods. Elise did not rank or assign primary status to either condoms or withdrawal when she used them together:

I: And why did you choose to do withdrawal and condoms at the same time?

R: Just because it’s just an extra. Like, what if the condom breaks? – Elise

FABMs were also used to buttress the efficacy of condoms. Vicky, who used condoms at every act of sex, described her contraceptive use like this:

I: Which of [these methods] have you used in this past year?

R: In the past year, just the male condom.

I: Just the male condom.

R: And then the calendar, to, like… reassure.

Vicky described how she used FABMs to determine when she was more fertile in order know when to be more vigilant about her partner’s condom use. During those times, she would monitor the way he put it on, make sure it stayed on throughout intercourse, and would examine the condom after sex by holding on to the rim and vigorously bouncing it around with ejaculate inside to make sure there were no holes or tears in it.

Women’s anxiety about the integrity of condoms was not the only motivation we observed for the practice of buttressing; this anxiety also often came from their partners. Alyssa identified condoms as her primary method of contraception, and reported using them at all of her sexual encounters over the past year. One of her partners did not trust condoms alone though:

I: Why did he use both [withdrawal and condoms]?

R: […]He always said, “I want to be careful, just in case. Sometimes condoms break.” That was his thinking. He was always kind of afraid of that, too.

Condoms were not the only less-effective method that was seen as requiring augmentation; our respondents also reported using withdrawal to buttress FABMs. Michele used withdrawal every time she had sex, but used FABMs to determine when she was most fertile so that she could abstain from sex on those days:

I: So, do you feel like [FABM is] a good method for you?

R: I think it’s okay. I think—I mean I would not rely on it solely. Ever. But I think it’s good. It’s a good way to kind of keep track of yourself and your body and what’s going on with it. […]

I: Have you ever had any problems remembering which days were safe?

R: Yes. But ultimately, I still use the withdrawal method every time. So, I feel confident that even if I don’t choose the right days, or even if we, like, have sex on the wrong days, then, you know, there’s always that second barrier.

Other women incorporated condoms into this mix; they buttressed FABMs by using withdrawal at acts of sex that occurred when their monitoring of their cycles indicated that they were at low risk of pregnancy, but when they had sex at a time that they determined that they were at higher risk, because they perceived condoms to be more effective than withdrawal.

The practice of buttressing hormonal methods with less-effective methods was also reported by our respondents. Condoms and withdrawal were the most commonly used buttresses for hormonal methods. Margery, for example, used her pill consistently. However, her fear of pregnancy was so strong that she felt the pill alone was not enough to protect her. Though her boyfriend did not want to use condoms, she insisted:

“…I don’t trust my body enough to, like, to be able to have sex without a condom– and not worry about it after. […] I’ll be paranoying [sic] from the date that we had sex all the way till my period. So, I–I really feel like I always need that second method.”

Mistrust of the efficacy of hormonal methods also motivated male partners to buttress them with other methods. Lucy used the IUD and personally felt adequately protected from pregnancy by this method. Her partner, however, was not convinced, and used withdrawal at every act of sex.

Women also buttressed methods when they could not be certain of their partner’s cooperation in the goal of not becoming pregnant. Amelia used FABMs buttressed by withdrawal at the beginning of the year, but switched to the injectable because her partner had stated that he wanted to impregnate her against her wishes. She was using the injectable and withdrawal simultaneously because her injectable use was clandestine; therefore, she needed to keep up the ruse that they were only using withdrawal for pregnancy protection. Yet, when asked if that was the only reason she used the two methods at the same time, she said:

“He didn’t know about it, and also I—you know, you can still get pregnant [on the injectable]. So I just wanted to just reassure myself that I was doing everything I could. […] Because, now I don’t trust any one method. So I use a multiple and combinations of everything that I can.”

Amelia’s explanation for buttressing of hormonal methods with withdrawal illustrated the permeable nature of her anxiety about the risk of unintended pregnancy; it was sparked by her partner’s attempts to thwart her contraceptive efforts, and was further informed by her mistrust of the efficacy of methods themselves.

FABMs were also used to buttress hormonal methods, despite the fact that hormonal contraception is intended to suppress ovulation, which ought to render FABMs moot. Lisa had been a consistent pill user for more than five years. However, she did not trust the pill completely to protect her from pregnancy, so she used FABMs to guide her in knowing which method to use in order to augment the pill’s effectiveness. On days when she perceived herself to be more fertile, she would use a condom. On days that she felt were safe, she would use withdrawal:

“…during the times when I have more or less possibility of getting pregnant, according to my period, we talk and we make a decision about it. For instance, today we might not use condoms because I know that this is…I don’t know, the week when I am less fertile. And, if it’s a week when I am more fertile, we’ll use condoms, with no exceptions.”

### Combining Mechanisms and Broad Method Mix

Lisa’s narrative helps illustrate how our respondents often utilized more than one mechanism of method combination. In the previous quote, Lisa states that she used condoms when she was fertile, with “no exceptions.” However, at another point in the interview, she described one instance in the past year of backing up her condom use with withdrawal when she felt her cycle indicated she should use a condom, but her partner did not have one. Therefore, Lisa (and 13 other respondents) was not just a “backer up” or a “buttresser,” but both.

A final example reiterates the flexibility of the practice of buttressing for our respondents (in that it did not always follow the hierarchy dictated by contraceptive methods’ published effectiveness rates) and the way that mechanisms can be combined. Jocelyn was an inconsistent pill user; she backed up her pill use with inconsistent condom use, which she then backed up with a tertiary method: withdrawal. However, when she was using both the pill and condoms simultaneously, Jocelyn not only thought of condoms as a backup for the pill, she also considered the pill as the buttress to condoms:

I: And what do you like about using the pill?

R: […] that it’ll prevent me from getting pregnant if ever I am to have a sexual encounter with someone and accidentally, the condom breaks.

In addition to using more than one mechanism of method combination, our respondents reported combining a wide range of methods in their attempts to protect themselves from unintended pregnancy (see Table 2). Our respondents described backing up strategies including: highly effective methods such as IUDs, the injectable, the patch, and the ring; methods with a high probability of user error such as pills, condoms, withdrawal, spermicides, and FABMs; and a method that is typically used as a backup, EC. They also utilized a method whose effectiveness defies classification: temporary celibacy. Among our 41 respondents who described either backing up or buttressing their methods, we recorded 15 different method combinations for backing up inconsistent method use, 16 different combinations for buttressing method effectiveness, and 13 method combinations that included both mechanisms.

**Table 2:** Combinations of methods used for backing up and buttressing

Backing up (15 combinations)		Buttressing (16 combinations)		Both—backing up buttressed combinations (13)	
Backing up one method with another	*N* of women	Hormonal with one LEM	*N* of women		*N* of women
*Backing up the pill*		Pill + withdrawal	6	EC for condoms/withdrawal/FABM	2
Withdrawal for pill	4	Pill + condoms	6	Withdrawal/FABM for condoms/FABM	2
EC for pill	2	IUD + withdrawal	2	Temp. celibacy for pill/FABM	1
Condoms for pill	2	Patch + condoms	1	Condoms/FABM for temp. celibacy	1
Temp. celibacy for pill	1	Patch + withdrawal	1	EC for withdrawal/FABM	1
Spermicides for pill	1	Injectable + condoms	1	Withdrawal/patch for condoms/patch	1
		Injectable + withdrawal	1	EC for condoms/FABM	1
*Backing up condoms*		Ring + condoms	1	Withdrawal/ring for condoms/ring	1
Withdrawal for condoms	5			Withdrawal/pill/FABM for condoms/pill/FABM	1
Temp. celibacy for condoms	1	Two LEMs		Withdrawal/spermicides for pill	1
		Withdrawal + FABM	6	EC for withdrawal/FABM	1
*Backing up withdrawal*		Condoms + FABM	2	Withdrawal for pill/FABM	1
Temp. celibacy for withdrawal	2			Withdrawal for injectable/condoms/FABM	1
EC for withdrawal	1	Three LEMs			
		Condoms + withdrawal + FABM	6		
*Backing up the injectable*		Condoms + withdrawal + spermicides	1		
Pill for injectable	1				
		Hormonals with two LEMs			
*Backing up spermicides*		Pill + withdrawal + FABM	3		
Withdrawal for spermicides	1	Pill + condoms + FAMB	2		
		Injectable + condoms + FABM	1		
“Tertiary” backing up					
Withdrawal for condoms for pill	3	Four LEMs			
EC for withdrawal for condoms	2	Condoms + withdrawal + spermicides + FABM	1		
Withdrawal for pill for injectable	1				
Withdrawal for temp. celibacy for injectable	1				

## Discussion

Our study sought to qualitatively examine the details of contraceptive use by obtaining women’s full contraceptive narratives from the past year among demographic subgroups of women known to experience the highest rates of contraceptive difficulties, unintended pregnancy, and abortion (Finer & Zolna, 2014; Frost & Darroch, 2008; Frost et al., 2007a; Jones et al., 2010; Mosher & Jones, 2010). Although we did not set out specifically to examine the practice of combination method use, it emerged as a nearly ubiquitous feature of our respondents’ contraceptive practices. We uncovered substantially more use of multiple methods in combination than has been documented in previous work, most of which finds less than a third of women to have used more than one method within a short timeframe (Brown et al., 2011; Frost et al., 2007b; Jones et al., 2014; Mosher & Jones, 2010; Parr & Siedlecky, 2007; Sato & Iwasawa, 2006; Spinelli et al., 2000; Toulemon & Leridon, 1998). In our sample, use of less-effective methods was also nearly universal, in contrast with national studies (Mosher & Jones, 2010).

Our data also expand our understanding of mechanisms of method combination. We found new reasons to substitute methods, new methods women felt that need to be backed up and that can be used as backups, and a new level of substitution than has previously been described. Earlier work on backing up inconsistent method use found respondents to be backing up missed pills (or pill use that may have become ineffective due to interactions with medication or illness) with condoms or EC (Abuabara et al., 2004; Gold, 1999; Gray et al., 1999; Horner et al., 2009; Oakley et al., 1997; Sanfilippo & Downing, 2008; Trussell & Guthrie, 2007; Trussell et al., 2014; Weisman et al., 1991; Whittaker et al., 2010; Williams-Deane & Potter, 1992). Our respondents also backed up missed pills with withdrawal, spermicides, and temporary celibacy, and backed up inconsistent use of the injectable. Other studies have documented method substitution between withdrawal and condoms (Jones et al., 2014) when condoms are unavailable, for male sexual pleasure, when intoxicated, due to lack of access to their primary method (e.g., running out of condoms or leaving home without their pills), and because of pressure from partners (Abuabara et al., 2004; Brown et al., 2011; Gray et al., 1999; Horner et al., 2009; Sanfilippo & Downing, 2008; Trussell & Guthrie, 2007; Trussell et al., 2014; Whittaker et al., 2010). We have added female sexual pleasure as a reason for such substitution (which is an aspect of women’s reproductive health behavior that remains understudied (Higgins & Hirsch, 2007)). Our respondents’ practices also indicated withdrawal, spermicides, and temporary celibacy to be methods that both required other methods to back them up and were used as backup to other coital methods. Finally, we uncovered an additional, tertiary level of backing up inconsistent method use, employed when respondents could not or did not use their usual backup method.

While some previous work has documented concurrent method use (Jones et al., 2014) and found that this process was enacted to augment the perceived efficacy of (usually less-effective) contraceptive methods (Arevalo et al., 2004; Gray et al., 1999; Guzman et al., 2013; Sinai & Jennings, 2014; Whittaker et al., 2010), we give this mechanism the name “buttressing,” and expand upon it in several ways. Jones et al. (2014) were surprised to find that some of their respondents used very highly effective methods such as IUDs concurrently with less-effective methods and questioned what could be motivating the practice; our data revealed that such combinations were often the result of buttressing. In fact, we found buttressing combinations involving highly effective methods to be just as common in our sample as those involving multiple less-effective methods detailed in previous work. The finding that male partners’ anxiety about method efficacy motivates buttressing was also echoed in our work (Gray et al., 1999; Whittaker et al., 2010). Pregnancy promoting behaviors from partners, as well as women’s personal concerns about their risk of pregnancy, also informed this practice. Finally, our data illustrated that the direction of augmentation in the practice of buttressing does not necessarily adhere to the standard hierarchy of method effectiveness, in that respondents often considered highly effective methods to be the buttress for less-effective methods.

The women in our sample described complex mechanisms of combining multiple layers of contraceptive methods in order to shield themselves from the risk of unintended pregnancy. A pervasive insecurity about both their own ability to prevent pregnancy through contraception and the efficacy of individual methods directly informed the complicated behaviors of backing up and buttressing contraceptive method use that we observed in our data. They were “always kind of afraid,” “paranoying [sic] from the date that we had sex all the way till my period,” and they used method on top of method “to reassure.” One of the main reasons that our respondents felt at risk of pregnancy despite their use of contraception was that they distrusted the efficacy of their methods. Previous work on perceptions of method efficacy has reached mixed conclusions: some studies find overestimation or accurate perceptions, but most work corroborates the underestimation of method efficacy that was prevalent in our sample (Biggs & Foster, 2013; Edwards, Oldman, Smith, McQuay, & Moore, 2000; Eisenberg et al., 2012; Frost, Lindberg, & Finer, 2012; Gray et al., 1999; Guzman et al., 2013; Horner et al., 2009; Murphy, Kirkman, & Hale, 1995; Tessler & Peipert, 1997; Whittaker et al., 2010). However, the connection between women’s understandings of the efficacy of their contraceptive methods and their actual use of those methods is difficult to determine. The one study that examined both perceptions of method efficacy and actual contraceptive use did not find any significant associations for women between underestimating the pills effectiveness and method use (Frost et al., 2012).

The connection between perceptions of efficacy and method use becomes even more opaque when considering less-effective methods. Our respondents reported combining methods because they were not confident about the efficacy of condoms, withdrawal, FABMs, and spermicides. Previous work has found that the same concerns have motivated combinations of less-effective methods (Biggs & Foster, 2013; Gray et al., 1999; Guzman et al., 2013; Horner et al., 2009; Murphy et al., 1995; Whittaker et al., 2010). The actual efficacy of most less-effective methods is difficult to gage for any individual, due to the large spread between the methods’ “perfect use” and “typical use” failure rates (Trussell, 2011). The actual failure rates of both withdrawal and FABMs are also a controversial issue among experts in the field (Arevalo et al., 2004; Doherty & Stuart, 2011; Guzman et al., 2013; Jones, Fennell, Higgins, & Blanchard, 2009; Killick, Leary, Trussell, & Guthrie, 2011; Trussell, 1995), and the effectiveness of temporary celibacy may not be possible to calculate (Dailard, 2003). Our respondents navigated this uncertainty by combining up to six different methods in combination through buttressing.

The majority of our respondents—despite the type of methods they used and consistency of with which they used them—felt vulnerable to unintended pregnancy. They spoke about not trusting themselves, not trusting their bodies, not trusting their partners, and not trusting their methods to effectively prevent pregnancy. This led them to layer multiple contraceptive methods on top of each other with a breadth and complexity that has not been documented in previous research. Notably, perceptions of susceptibility to unintended pregnancy have been studied before, but mainly from the opposite direction; perceptions of a *lack* of susceptibility have been posited and, in some studies, documented as a contributing factor to the *nonuse* of contraception (Biggs & Foster, 2013; Breheny & Stephens, 2004; Frohwirth, Moore, & Maniaci, 2013; Nettleman, Chung, Brewer, Ayoola, & Reed, 2007; Polis & Zabin, 2012; Rahman, Berenson, & Herrera, 2013; Reed et al., 2014). In contrast, our study documents a clear relationship between the perception of increased susceptibility to unintended pregnancy and concurrent combination method use.

### Strengths and Limitations

Our findings must be viewed in light of the fact that they rely on women’s self-reports and retrospective recall. However, in contrast to other qualitative studies that have attempted to examine detailed narratives of contraceptive use (Horner et al., 2009; Reed et al., 2014; Whittaker et al., 2010), the current study focused only on the past year, and procedures and questions were geared toward drawing out and clarifying every detail of women’s contraceptive behavior during the timeframe. Another limitation concerns the applicability of our findings to women in general. Small qualitative samples are not designed to produce generalizable findings, especially given that our sample was selected in order to elucidate the practices of women most at risk of unintended pregnancy (Finer & Zolna, 2014; Frost & Darroch, 2008; Frost et al., 2007a; Jones et al., 2010; Mosher & Jones, 2010). For these two reasons, it is possible that these results may overstate the prevalence of combination method use for the rest of the population. Additionally, our respondents may have over-reported their method use due to social desirability bias (Stuart & Grimes, 2009). We attempted to mitigate the influence of this effect through techniques such as using language that acknowledged and normalized method use that is perceived as “incorrect” (e.g., removing condoms during sex) and ineffective (probing on methods such as withdrawal and FABMs), as well as contraceptive nonuse.

### Implications

When Gray et al. (1999) discuss the patterns of contraceptive use they saw in their data from Bangladesh, they conclude that it is the combination, rather than the individual methods “that is really the method being used” (p. 51). Our data show that this conclusion may be equally applicable to women in the U.S. at risk of unintended pregnancy, and therefore should be incorporated into contraceptive counseling practice. There are several ways that this could be achieved. Our respondents perceived that their concurrent combination method use, which often comprised less-effective methods, augmented their level of protection from pregnancy as compared to using only one of these methods. Counselors and other health professionals need to be aware of this belief so that they can uncover women’s combination method use and help users assess whether these behaviors are actually affording them the level of protection they are seeking. Contraceptive counseling can also take combination method use into account when advising women about switching methods. Previous work shows a connection between switching methods and exposure to unintended pregnancy (Frost et al., 2007b), and our data make clear that for many women this switching may not be from just one method to another, but may involve combinations of methods. Contraceptive counseling guidelines also advise that women who wish to switch methods should overlap methods to avoid a gap in coverage (Centers for Disease Control and Prevention, 2013); acknowledging concurrent method use may make this recommendation easier to follow.

Women using contraceptive methods care about efficacy, but that valuation does not always translate into use of one highly effective method. Current clinical practice tends to focus on a tiered approach highlighting the most-effective methods up front, particularly the IUD and implant (Gavin et al., 2014; Madden, Mullersman, Omvig, Secura, & Peipert, 2013; Trussell et al., 2013). It is clear why the public health model makes this recommendation; from a rationalist perspective, the insecurity prompted by inconsistent method use and ineffective methods that our respondents experienced could be ameliorated by switching to long-acting reversible contraceptives (which a few respondents in the sample did). Yet women who decline to use these highly effective methods may still benefit from counseling about ways to increase their level of protection from pregnancy by engaging in contraceptive practices using less-effective methods (Luker, 1999). Finally, because respondents who were using methods that are considered moderately to highly effective expressed doubt that they were adequately protected, counseling can acknowledge these beliefs while addressing women’s concerns.

These findings have implications for research on contraceptive use as well as counseling. Jones et al. discussed the reasons for underreporting of withdrawal, and their survey design innovation shows that reporting can be improved (Jones et al., 2014). Our results indicate that other less-effective methods such as condoms, FABMs, EC, spermicides, and even temporary celibacy may be similarly underreported; further research could test whether reporting of use of these methods could be increased if survey design changes. Our findings also indicate that research based on large-scale national survey reporting of contraceptive use that utilizes only the “most-effective method used” variables, while useful in understanding the risk of unintended pregnancy at the population level, can miss complex contraceptive behavior. Analyses that take concurrent multiple method use into account will better reflect women’s actual contraceptive practices. Our findings show that qualitative or mixed-methods approaches have great value in this endeavor. Finally, while our data revealed that women are often operating under assumptions about the efficacy and effects of methods that are not factual, it was beyond the scope to this study to fully examine the content and origin of those beliefs. This would be a rich area for subsequent exploration.

Examining how women use methods in combination provides insight into the effort they put into preventing pregnancy, as well as illuminating the beliefs and motivations that underlie that behavior. Taken together, our results (and the other work examining multiple method use that we build on) illustrate that women may not only be contraceptive “risk-takers” or cost-benefit analysts (Luker, 1977), fatalists (Woodsong, Shedlin, & Koo, 2004), and “magical-thinkers,” (Frohwirth et al., 2013), but are also extremely anxious about unintended pregnancy, and highly motivated by the desire to avoid that outcome. Contraceptive counseling (as well as the theory and research that inform it) that acknowledges women’s practices of and motivations for multiple method use that may result, might better resonate with women’s lived experiences.

## Footnotes

1 The designation of “less-effective” (including withdrawal, fertility-awareness-based and barrier methods) is derived from calculations by Trussell (2011) on typical-use rates of contraceptive failure, and is widely reflected in clinical practice. “Highly-effective” methods include long-acting reversible methods, the injectable, the pill, the patch, and the ring.

2 Meaning, the method that women considered the principal way they were contracepting, usually defined as the method they used most often with a particular partner to prevent pregnancy.

3 Middle-income was defined as 200–300 % of the federal poverty level; lower-income was defined as below 200 % of the federal poverty level.

## References

[CR1] Abuabara K, Becker D, Ellertson C, Blanchard K, Schiavon R, Garcia SG (2004). As often as needed: Appropriate use of emergency contraceptive pills. Contraception.

[CR2] Agar MH (1980). The professional stranger: An informal introduction to ethnography.

[CR3] Arevalo M, Jennings V, Nikula M, Sinai I (2004). Efficacy of the new TwoDay Method of family planning. Fertility and Sterility.

[CR4] Berer M (2006). Dual protection: More needed than practiced or understood. Reproductive Health Matters.

[CR5] Biggs MA, Foster DG (2013). Misunderstanding the risk of conception from unprotected and protected sex. Women’s Health Issues.

[CR6] Breheny M, Stephens C (2004). Barriers to effective contraception and strategies for overcoming them among adolescent mothers. Public Health Nursing.

[CR7] Brown, J., Hennessy, M., Sales, J., DiClemente, R., Salazar, L., Vanable, P., … Stanton, B. (2011). Multiple method contraception use among African American adolescents in four US cities. *Infectious Diseases in Obstetrics and Gynecology*. doi:10.1155/2011/765917. 10.1155/2011/765917 PMC3139861 21785557

[CR8] Centers for Disease Control and Prevention (2013). U.S. selected practice recommendations for contraceptive use, 2013. Adapted from the World Health Organization selected practice recommendations for contraceptive use.

[CR9] Dailard, C. (2003). Understanding ‘abstinence’: Implications for individuals, programs and policies. *The Guttmacher Report on Public Policy, 6*(5), 4–6. Retrieved from https://www.guttmacher.org/pubs/tgr/06/5/gr060504.html.

[CR10] Doherty IA, Stuart GS (2011). Coitus interruptus is not contraception. Sexually Transmitted Diseases.

[CR11] Edwards JE, Oldman A, Smith L, McQuay HJ, Moore RA (2000). Women’s knowledge of, and attitudes to, contraceptive effectiveness and adverse health effects. British Journal of Family Planning.

[CR12] Eisenberg DL, Allsworth JE, Zhao Q, Peipert JF (2012). Correlates of dual-method contraceptive use: An analysis of the National Survey of Family Growth (2006–2008). Infectious Diseases in Obstetrics and Gynecology.

[CR13] Eisenberg DL, Secura GM, Madden TE, Allsworth JE, Zhao Q, Peipert JF (2012). Knowledge of contraceptive effectiveness. American Journal of Obstetrics and Gynecology.

[CR14] Finer LB, Zolna MR (2014). Shifts in intended and unintended pregnancies in the United States, 2001–2008. American Journal of Public Health.

[CR15] Frohwirth L, Moore AM, Maniaci R (2013). Perceptions of susceptibility to pregnancy among U.S. women obtaining abortions. Social Science and Medicine.

[CR16] Frost JJ, Darroch JE (2008). Factors associated with contraceptive choice and inconsistent method use, United States, 2004. Perspectives on Sexual and Reproductive Health.

[CR17] Frost JJ, Lindberg LD, Finer LB (2012). Young adults’ contraceptive knowledge, norms and attitudes: Associations with risk of unintended pregnancy. Perspectives on Sexual and Reproductive Health.

[CR18] Frost JJ, Singh S, Finer LB (2007). Factors associated with contraceptive use and nonuse, United States, 2004. Perspectives on Sexual and Reproductive Health.

[CR19] Frost JJ, Singh S, Finer LB (2007). U.S. women’s one-year contraceptive use patterns, 2004. Perspectives on Sexual and Reproductive Health.

[CR20] Gavin, L., Moskosky, S., Carter, M., Curtis, K., Glass, E., Godfrey, E., … Zapata, L. (2014). Providing quality family planning services: Recommendations of CDC and the U.S. Office of Population Affairs. *MMWR Recommendations and Reports, 63*(RR-04), 1–54. 24759690

[CR21] Gold MA (1999). Prescribing and managing oral contraceptive pills and emergency contraception for adolescents. Pediatric Clinics of North America.

[CR22] Grady WR, Billy JOG, Klepinger DH (2002). Contraceptive method switching in the United States. Perspectives on Sexual and Reproductive Health.

[CR23] Gray A, Chowdhury JH, Caldwell B, Al-Sabir A (1999). Coitus-dependent family planning methods: Observations from Bangladesh. Studies in Family Planning.

[CR24] Guzman L, Caal S, Peterson K, Ramos M, Hickman S (2013). The use of fertility awareness methods (FAM) among young adult Latina and black women: What do they know and how well do they use it? Use of FAM among Latina and Black women in the United States. Contraception.

[CR25] Higgins JA, Cooper AD (2012). Dual use of condoms and contraceptives in the USA. Sexual Health.

[CR26] Higgins JA, Hirsch JS (2007). The pleasure deficit: Revisiting the “sexuality connection” in reproductive health. Perspectives on Sexual and Reproductive Health.

[CR27] Horner, J. R., Salazar, L. F., Romer, D., Vanable, P. A., DiClemente, R., Carey, M. P., et al. (2009). Withdrawal (coitus interruptus) as a sexual risk reduction strategy: Perspectives from African-American adolescents. *Archives of Sexual Behavior, 38*, 779–787. doi:10.1007/s10508-007-9304-y. 10.1007/s10508-007-9304-y PMC4218729 18293076

[CR28] Huberman AM, Miles MB, Denzin NK, Lincoln YS (1998). Data management and analysis methods. Handbook of qualitative research.

[CR29] Jaccard J (2009). Unlocking the contraception conundrum: Reducing unplanned pregnancies in emerging adulthood.

[CR30] Jennings V, Burke A, Hatcher R, Trussell J, Nelson A, Cates W, Kowal D, Policar M (2011). Fertility awareness-based methods. Contraceptive technology.

[CR31] Jones RK, Fennell J, Higgins JA, Blanchard K (2009). Better than nothing or savvy risk-reduction practice? The importance of withdrawal. Contraception.

[CR32] Jones RK, Finer LB, Singh S (2010). Characteristics of U.S. abortion patients, 2008.

[CR33] Jones RK, Lindberg LD, Higgins JA (2014). Pull and pray or extra protection? Contraceptive strategies involving withdrawal among US adult women. Contraception.

[CR34] Jones J, Mosher W, Daniels K (2012). Current contraceptive use in the United States, 2006–2010, and changes in patterns of use since 1995 (60).

[CR35] Keogh LA (2005). A qualitative study of women’s use of emergency contraception. Journal of Family Planning and Reproductive Health Care.

[CR36] Killick SR, Leary C, Trussell J, Guthrie KA (2011). Sperm content of pre-ejaculatory fluid. Human Fertility.

[CR37] Lansky A, Thomas JC, Earp JA (1998). Partner-specific sexual behaviors among persons with both main and other partners. Family Planning Perspectives.

[CR38] LeCompte MD, Schensul JJ (1999). Analyzing and interpreting ethnographic data.

[CR39] Lescano, C. M., Vazquez, E. A., Brown, L. K., Litvin, E. B., Pugatch, D., & Project SHIELD Study Group. (2006). Condom use with “casual” and “main” partners: What’s in a name? *Journal of Adolescent Health, 39*, 443.e1–443.e7. doi:10.1016/j.jadohealth.2006.01.003. 10.1016/j.jadohealth.2006.01.003 16919809

[CR40] Luker K (1977). Contraceptive risk taking and abortion: Results and implications of a San Francisco Bay Area study. Studies in Family Planning.

[CR41] Luker KC (1999). A reminder that human behavior frequently refuses to conform to models created by researchers. Family Planning Perspectives.

[CR42] Macaluso M, Demand MJ, Artz LM, Hook EW (2000). Partner type and condom use. AIDS.

[CR43] Madden T, Mullersman JL, Omvig KJ, Secura GM, Peipert JF (2013). Structured contraceptive counseling provided by the Contraceptive CHOICE Project. Contraception.

[CR44] Migration Policy Institute. (2014). *Migration Policy Institute tabulation of data from the U.S. Census Bureau’s 2010 and 2012 American Community Surveys, and 2000 Decennial Census*. Retrieved from http://www.migrationpolicy.org/programs/data-hub/us-immigration-trends#source.

[CR45] Miles MB, Huberman AM (1994). Qualitative data analysis: An expanded sourcebook.

[CR46] Mosher, W. D., & Jones, J. (2010). *Use of contraception in the United States: 1982*–*2008* (23). Retrieved from http://www.cdc.gov/nchs/data/series/sr_23/sr23_029.pdf. 20939159

[CR47] Murphy P, Kirkman A, Hale RW (1995). A national survey of women’s attitudes toward oral contraception and other forms of birth control. Women’s Health Issues.

[CR48] Nettleman MD, Chung H, Brewer J, Ayoola A, Reed PL (2007). Reasons for unprotected intercourse: Analysis of the PRAMS survey. Contraception.

[CR49] Oakley, D., Potter, L., de Leon-Wong, E., & Visness, C. (1997). Oral contraceptive use and protective behavior after missed pills. *Family Planning Perspectives,**29*, 277–279, 287. doi:10.2307/2953417. 9429874

[CR50] Parr N, Siedlecky S (2007). Use of ‘dual protection’ and other combinations of contraceptive methods in Australia. Australian and New Zealand Journal of Public Health.

[CR51] Polis CB, Zabin LS (2012). Missed conceptions or misconceptions: Perceived infertility among unmarried young adults in the United States. Perspectives on Sexual and Reproductive Health.

[CR52] Rahman M, Berenson AB, Herrera SR (2013). Perceived susceptibility to pregnancy and its association with safer sex, contraceptive adherence, and subsequent pregnancy among adolescent and young adult women. Contraception.

[CR53] Reed J, England P, Littlejohn K, Conroy Bass B, Caudillo ML (2014). Consistent and inconsistent contraception among young women: Insights from qualitative interviews. Family Relations.

[CR54] Rosenberg MJ, Waugh MS (1998). Oral contraceptive discontinuation: A prospective evaluation of frequency and reasons. American Journal of Obstetrics and Gynecology.

[CR55] Sanfilippo J, Downing D (2008). Emergency contraception: When and how to use it. Journal of Family Practice.

[CR56] Sato, R., & Iwasawa, M. (2006). Contraceptive use and induced abortion in Japan: How is it so unique among the developed countries. *Japanese Journal of Population, 4*, 33–54. Retrieved from https://www.researchgate.net/publication/228502968.

[CR57] Sinai, I., & Jennings, V. (2014). Response to Guzman et al.: The use of fertility awareness methods (FAM) among young adult Latina and black women. *Contraception, 89*, 67. doi:10.1016/j.contraception.2013.09.002. 10.1016/j.contraception.2013.09.002 24125123

[CR58] Spinelli A, Talamanca IF, Lauria L, European Study Group on Infertility and Subfecundity (2000). Patterns of contraceptive use in 5 European countries. American Journal of Public Health.

[CR59] Stuart GS, Grimes DA (2009). Social desirability bias in family planning studies: A neglected problem. Contraception.

[CR60] Sznitman, S. R., Romer, D., Brown, L. K., DiClemente, R. J., Valois, R. F., Vanable, P. A., … Stanton, B. (2009). Prevalence, correlates, and sexually transmitted infection risk related to coitus interruptus among African-American adolescents. *Sexually Transmitted Diseases, 36*, 218–220. doi:10.1097/OLQ.0b013e3181901c8f. 10.1097/OLQ.0b013e3181901c8f PMC2709749 19265743

[CR61] Tessler SL, Peipert JF (1997). Perceptions of contraceptive effectiveness and health effects of oral contraception. Women’s Health Issues.

[CR62] Toulemon, L., & Leridon, H. (1998). Contraceptive practices and trends in France. *Family Planning Perspectives, 30*, 114–120. Retrieved from http://www.guttmacher.org/pubs/journals/3011498.html. 9635259

[CR63] Trussell J (1995). On the efficacy of withdrawal. Studies in Family Planning.

[CR64] Trussell J (2011). Contraceptive failure in the United States. Contraception.

[CR65] Trussell J, Guthrie KA (2007). Talking straight about emergency contraception. Journal of Family Planning and Reproductive Health Care.

[CR66] Trussell J, Henry N, Hassan F, Prezioso A, Law A, Filonenko A (2013). Burden of unintended pregnancy in the United States: Potential savings with increased use of long-acting reversible contraception. Contraception.

[CR67] Trussell J, Raymond EG, Cleland K (2014). Emergency contraception: A last chance to prevent unintended pregnancy. Contemporary Readings in Law and Social Justice.

[CR68] Tyler, C., Whiteman, M., Kraft, J., Zapata, L., Hillis, S., Curtis, K., … Marchbanks, P. A. (2014). Dual use of condoms with other contraceptive methods among adolescents and young women in the United States. *Journal of Adolescent Health, 54*, 169–175. 10.1016/j.jadohealth.2013.07.042 24074606

[CR69] Vaughan B, Trussell J, Kost K, Singh S, Jones RK (2008). Discontinuation and resumption of contraceptive use: Results from the 2002 National Survey of Family Growth. Contraception.

[CR70] Weisman CS, Plictha S, Nathanson CA, Ensminger M, Robinson JC (1991). Condoms for disease prevention among adolescent users of oral contraceptives. Family Planning Perspectives.

[CR71] Wellings, K., Brima, N., Sadler, K., Copas, A. J., McDaid, L., Mercer, C. H., … Glasier, A. (2015). Stopping and switching contraceptive methods: Findings from Contessa, a prospective longitudinal study of women of reproductive age in England. *Contraception, 91*, 57–66. 10.1016/j.contraception.2014.09.008 25444254

[CR72] Whittaker PG, Merkh RD, Henry-Moss D, Hock-Long L (2010). Withdrawal attitudes and experiences: A qualitative perspective among young urban adults. Perspectives on Sexual and Reproductive Health.

[CR73] Williams-Deane M, Potter LS (1992). Oral contraceptive use instructions: An analysis of patient package inserts. Family Planning Perspectives.

[CR74] Woodsong C, Koo HP (1999). Two good reasons: Women’s and men’s perspectives on dual contraceptive use. Social Science and Medicine.

[CR75] Woodsong C, Shedlin M, Koo H (2004). The ‘natural’ body, God and contraceptive use in the southeastern United States. Culture Health and Sexuality.

